# A Case of Fibrinous Pericardial Effusion: Masquerading Presentation of Systemic Lupus Erythematosus

**DOI:** 10.7759/cureus.40917

**Published:** 2023-06-25

**Authors:** Kamal Joshi, Aman Elwadhi, Binita Poudel, Anukriti Agnihotry, Prashant K Verma

**Affiliations:** 1 Pediatrics, All India Institute of Medical Sciences, Rishikesh, IND

**Keywords:** low and middle country (lmic), ada, lupus nephritis, fibrinous pericarditis, tuberculosis, sle

## Abstract

Systemic lupus erythematosus (SLE) is a multisystem autoimmune disorder with varied presentations varying from nonspecific features like fever, malaise, and arthralgia to serious manifestations like serositis (pleural, pericardial effusions), neurological manifestations, and renal involvement (lupus nephritis). SLE is a great mimicker, especially for infections like tuberculosis (TB) which is rampant in low- and middle-income countries (LMIC). We report a case of massive pericardial effusion, which was initially diagnosed as TB on clinico-radiological basis, but the diagnosis was later revised owing to new findings.

## Introduction

Systemic lupus erythematosus (SLE) is a chronic autoimmune disorder with varied presentations due to its effect on multiple organ systems [1]. It occurs due to a complex interaction of impaired apoptotic clearance, upregulation of innate and adaptive immune systems, complement activation, immune complexes, and tissue inflammation culminating in an autoimmune process [1]. Non-specific features like fever, malaise, and arthralgias are the common presentations. Mucocutaneous involvement is seen in more than 80% of the patients [2]. However, it may present with some serious manifestations like serositis (pleural, pericardial effusions), neurological manifestations, and renal involvement (leading to lupus nephritis). Due to its varied presentations, it mimics other conditions with multisystemic involvement, like tuberculosis and other connective tissue disorders leading to misdiagnosis and mismanagement, adding to morbidity and mortality of the disease. In this article, we present a unique case of fibrinous pericarditis, which initially showed features of tuberculosis (TB) clinico-radiologically, but later turned out to be SLE.

## Case presentation

**Figure 1:**
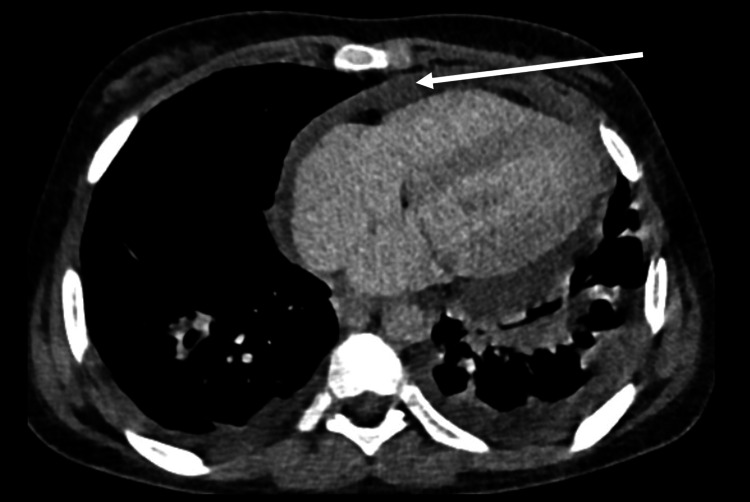
Contrast-enhanced computed tomography (CECT) chest axial cuts showing pericardial effusion (white arrow)

A 15-year-old girl with premorbid normal status, completely immunized with bacille Calmette-Guerin (BCG) scar present over the left deltoid region, presented with complaints of low-grade undocumented fever, dyspnea, and pleuritic chest pain for two months. There was no history of TB contact, cough, joint pain, rash, or photosensitivity. Chest radiograph and contrast-enhanced computed tomography (CECT) chest showed cardiomegaly (Figure 1) with an electrocardiogram (ECG) showing decreased amplitudes of QRS complex and T wave. Echocardiography showed massive pericardial effusion with fibrinous strands. 

Pericardiocentesis was done, which revealed serosanguinous fluid with elevated white blood cells (27,200 cells/µL, lymphocyte 70%) with raised adenosine deaminase levels (ADA) (193 IU/L). However, acid-fast bacilli staining was negative. Cartridge-based nucleic acid amplification test (CBNAAT) was not done due to logistic reasons. Considering the epidemiological (high prevalence of tuberculosis in the community), clinical (chronic history, constitutional symptoms, and positive tuberculin sensitivity test), and laboratory evidence (lymphocytic pleocytosis and elevated ADA), tubercular pericardial effusion was the foremost differential, and the patient was started on empirical anti-tubercular therapy (ATT) as per the directly observed therapy short course (DOTS) regime (two months of isoniazid, rifampicin, ethambutol, and pyrazinamide followed by four months of isoniazid, rifampicin, ethambutol) along with oral steroids and kept under vigilant follow-up. 

The patient showed initial clinical and radiological improvement following therapy for 1.5 months, but as steroids were tapered and stopped, the symptoms recurred in the form of fever and breathlessness within two months of the stoppage of steroids in addition to new complaints of arthritis of small and large joints and anasarca. At this point, possibilities of multidrug-resistant TB and connective tissue disorders like SLE were considered. Investigations revealed normocytic normochromic anemia (hemoglobin 8.2 gm/dl), leukopenia (2.9 x 109cells/L), elevated erythrocyte sedimentation rate (ESR 85mm/h), and C-reactive protein (CRP 31 mg/L), proteinuria (24-hour urine protein - 2 gram) with microscopic hematuria with normal renal profile and reappearance of pericardial effusion. 

**Figure 2:**
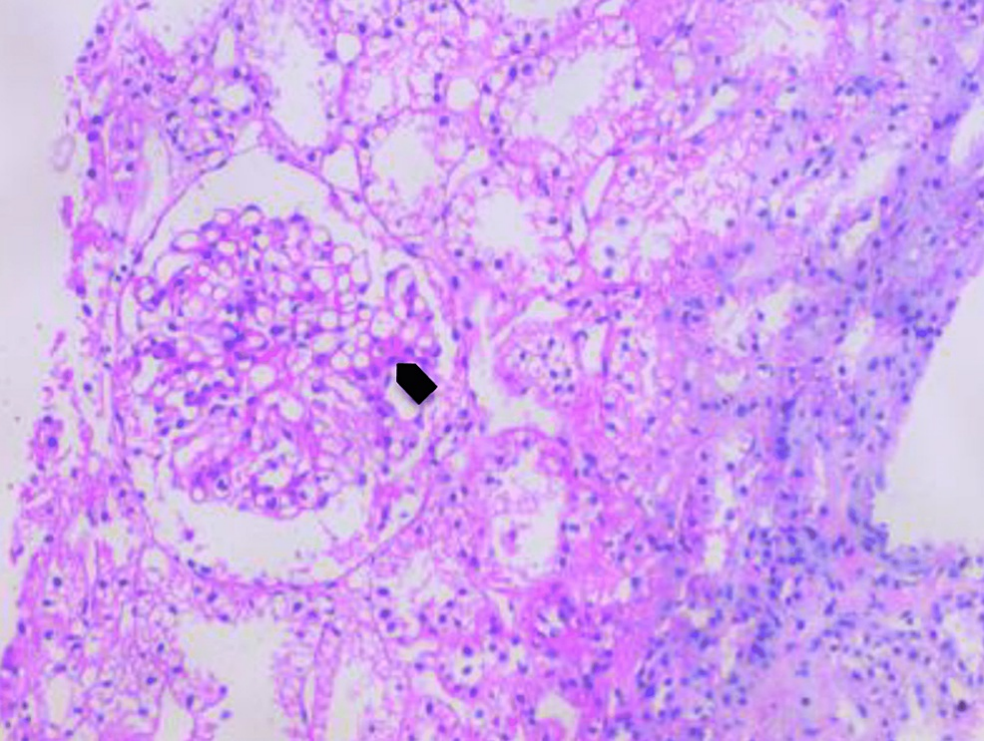
Renal biopsy (40x, Hematoxylin and Eosin stain) showing endocapillary proliferation, increase in the mesangial matrix (black arrow), and increased cellularity

Etiological investigations revealed positive tests for antinuclear antibody (ANA - patchy granular pattern) and anti-double-stranded DNA (dsDNA). For confirmation of lupus nephritis, a renal biopsy was done, which revealed features of membranous (class V) and focal lupus nephritis (class III) with tissue ANA phenomena (Figure 2). Hence a diagnosis of SLE with lupus nephritis (class V/III) was made, and the patient was started on immunosuppressive treatment as per National Institutes of Health (NIH) protocol with induction phase of steroids and monthly cyclophosphamide therapy and stoppage of ATT. Following treatment, the patient's condition gradually improved with the resolution of arthritis, nephritis, pericardial effusion, and cytopenia. The child is presently in the maintenance phase and under regular follow-up, tolerating treatment well with no adverse effects to date and without any flare-ups in the last six months.

## Discussion

In this case report, we describe a case of SLE masquerading as tuberculosis. During the initial presentation, the patient exhibited significant pericardial effusion with high adenosine deaminase levels. There were no other features suggestive of systemic lupus erythematosus. Due to the high prevalence of tuberculosis in LMIC and the presence of pericardial effusion with fibrinous strands and elevated ADA levels, the patient was suspected to have tuberculosis. The patient's second presentation included multiple system involvement, ANA positivity, and renal biopsy findings consistent with lupus nephritis, fulfilling the 2019 European League Against Rheumatism/American College of Rheumatology (EULAR/ACR) Classification Criteria for SLE. Hence a revised diagnosis of SLE with lupus nephritis was made.

Raised ADA level is not specific to tuberculosis and can be seen in conditions causing cellular system activation like typhoid, brucellosis, connective tissue disorders (SLE, rheumatoid arthritis), malignancies, liver disease, and sarcoidosis [3]. An increase in ADA levels should not be the sole criterion to start antitubercular therapy. Our patient had an atypical presentation of SLE, making the diagnosis challenging. Diagnosis of SLE should be borne in mind when an adolescent girl presents with multisystem involvement.

The treatment armamentarium includes immunosuppressive drugs like steroids, cyclophosphamide, and mycophenolate mofetil [4]. In the presence of active organ involvement like lupus nephritis, a higher level of treatment is indicated. The management protocol of lupus nephritis given by ACR is known as NIH protocol [5]. No immunosuppressive therapy is required for class I and II lupus nephritis, whereas class III and IV nephritis require aggressive therapy with glucocorticoid and immunosuppressive therapy. Class V, in combination with class III or class IV, should be treated in the same manner as III or IV alone. Pure class V requires low-dose prednisolone (0.5mg/kg/day) with mycophenolate mofetil. Class VI requires renal replacement therapy instead of immunosuppressive therapy [5].

Our patient had Class III lupus nephritis in combination with class V nephritis. The treatment, according to NIH protocol, involves the induction phase using monthly cyclophosphamide or mycophenolate mofetil along with oral and/or iv glucocorticoid therapy and the maintenance phase with mycophenolate mofetil and azathioprine. Other protocols, including the the Euro-Lupus protocol [6] using low-dose cyclophosphamide, are yet to be studied in children.

## Conclusions

SLE and TB are common diseases in LMIC which can have similar presentations. Both are common causes of fibrinous pericarditis. Epidemiologically, as TB is more common, it is overdiagnosed. However, other differentials, including connective tissue disorders, should be kept in mind. SLE, if not diagnosed early and treated appropriately, may lead to severe morbidity and mortality. Hereby, we opine that any child with serositis should be thoroughly investigated for underlying connective tissue disorders beyond common infections.
